# Ovalbumin Epitope SIINFEKL Self-Assembles into a Supramolecular Hydrogel

**DOI:** 10.1038/s41598-019-39148-8

**Published:** 2019-02-25

**Authors:** Meder Kamalov, Hanspeter Kählig, Christian Rentenberger, Alexander R.M. Müllner, Herwig Peterlik, Christian F. W. Becker

**Affiliations:** 1 Institute of Biological Chemistry, Faculty of Chemistry, University of Vienna, Währinger Strasse 38, 1090 Vienna, Austria; 2 Institute of Organic Chemistry, Faculty of Chemistry, University of Vienna, Währinger Strasse 38, 1090 Vienna, Austria; 3 Physics of Nanostructured Materials, Faculty of Physics, University of Vienna, Boltzmanngasse 5, 1090 Vienna, Austria; 4 Dynamics of Condensed Systems, Faculty of Physics, University of Vienna, Boltzmanngasse 5, 1090 Vienna, Austria

## Abstract

Here we show that the well-known ovalbumin epitope SIINFEKL that is routinely used to stimulate ovalbumin-specific T cells and to test new vaccine adjuvants can form a stable hydrogel. We investigate properties of this hydrogel by a range of spectroscopic and imaging techniques demonstrating that the hydrogel is stabilized by self-assembly of the peptide into nanofibres via stacking of β-sheets. As peptide hydrogels are known to stimulate an immune response as adjuvants, the immunoactive properties of the SIINFEKL peptide may also originate from its propensity to self-assemble into a hydrogel. This finding requires a re-evaluation of this epitope in adjuvant testing.

## Introduction

Fibrillar nanostructures can be assembled by a number of natural and designed peptides, which usually possess distinct biophysical properties, such as propensities for β-sheet folds. Nanofibrils can also form as a result of protein or peptide misfolding, which is associated, for example, with pathogeneses of neurodegenerative disorders such as Parkinson’s and Alzheimer’s diseases^1^. In biological settings, peptide nanofibrils can have important functional roles, the extracellular amyloid fibrils of *E. coli*, for example, are used for cell propulsion^2^. More recently, it has been possible to tune and employ the fibril-forming properties of peptides in the design and manufacture of functional nanomaterials that can be used in disease treatment and prevention^3,4^. Self-assembled peptides in this case possess several advantages, which include multi-valency, defined synthetic composition, tuned specificity, and ease of further functionalisations^5,6^. These advantages have allowed peptide nanofibrils, as well as the associated hydrogels, to be successfully used as scaffolds in regenerative medicine, cell culture matrices and vehicles for drug delivery^7,8^. Certain peptides that constitute fragments of full-length proteins have been described to form fibrils and hydrogels. These include mouse laminin a-1^9^, human transthyretin^10^ and human troponin C^11^. Peptide fragments of each of these proteins assemble into nanofibrils networks *in vitro*, with further assembly leading to formation of stiff hydrogels.

A new and exciting application of fibrillar peptide assemblies is in adjuvanting of subunit vaccines^12^. Although subunit vaccines show remarkable promise in treatment and prevention of deadly diseases, they suffer from poor immunogenicity, which substantially limits their efficacies^13^. Adjuvanting or enhancing the immunogenicities of subunit vaccines is therefore a significant challenge in biomedical research. A range of materials is currently under development as vaccine adjuvants and among them are hydrogels consisting of peptide nanofibrils. Peptides in this case can be conjugated to specific antigens or epitopes, often themselves peptides, and subsequently used in targeted immunisations^14–17^. The disease antigens and individual peptide epitopes are identified through an analysis of a given immune response against an immunogen, which lead to the individual peptide fragments that directly interact with the cells of the immune system^18^. Some of these peptide epitopes are capable of eliciting an immune response in the absence of the parent pathogen but such activity is heavily dependent on the addition of proper adjuvants^19^.

During the development of adjuvants, well-known and well-characterised peptide antigens are used to evaluate the adjuvant efficacies. Ovalbumin (OVA) has been historically a popular source of such antigens, since OVA can induce both humoral and cellular immune responses based on well-characterised peptide epitopes^20,21^. The OVA_257-264_ octapeptide was one of the first OVA epitopes to be characterised, it has an amino acid sequence SIINFEKL, which is recognised by cytotoxic T lymphocytes^18^. Immunisation with the adjuvanted SIINFEKL peptide induces long-lasting CD8+ T cell immunity in mice^22^.

Here we report on the properties of the OVA epitope SIINFEKL to self-assemble into fibrillar nanostructures that lead to formation of a peptide hydrogel. The properties of this peptide assembly have been analysed by use of rheology, electron microscopy, small angle X-ray scattering (SAXS), circular dichroism (CD) and infrared (IR) spectroscopies. The molecular analysis of the peptide fold has been carried out with peptide nuclear magnetic resonance (NMR) measurements. It is demonstrated that SIINFEKL forms fibrillar assemblies similar to other peptide hydrogels. The immunoactive properties of this peptide can therefore be related to its self-assembling nature.

## Results and Discussion

**Figure 1:**
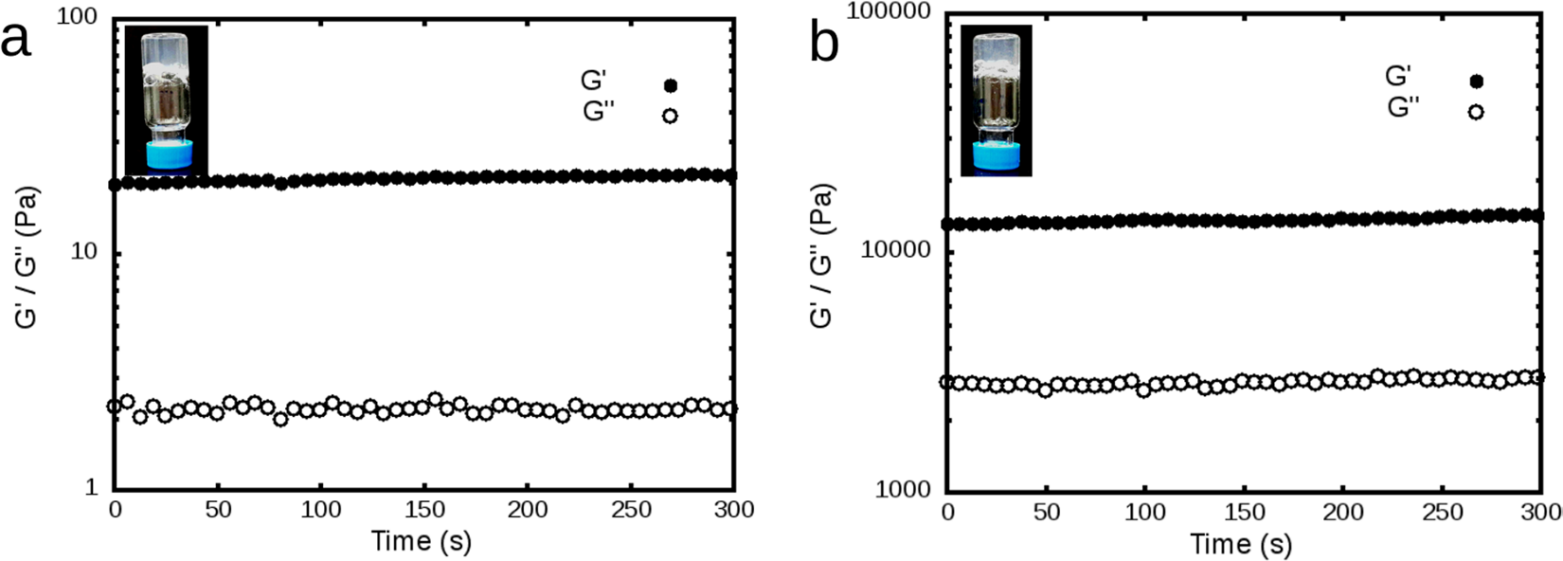
Hydrogel vials following overnight incubation and the corresponding rheological sweep data on 0.5% (**a**) and 1% (**b**) SIINFEKL hydrogel. With Y-axis in log scale, significant increase in both G’ and G” values for the 1% hydrogel can be seen.

The OVA_257-264_ octapeptide SIINFEKL was prepared via standard Fmoc-SPPS and purified via HPLC (Fig. SI-1). Gel formation was observable first during precipitation of this peptide in diethyl ether following cleavage with trifluoroacetic acid (TFA). The gel forming property was confirmed by preparing a 1% (w/v) solution of the purified peptide in Millipore water, where the gel formed immediately after dissolving the peptide (Fig. 1a). A hydrogel also forms with 0.5% peptide in water following overnight incubation. A typical peptide concentration used to prepare peptide-based vaccines is 8 mM^14–16^ and the case of SIINFEKL the concentration of 8 mM would amount to 7.7 mg/ml or 0.77% (w/v) solution, which is well within the gelation conditions.

The mechanical properties of the SIINFEKL hydrogel at these two concentrations were evaluated by use of rheology after incubation at room temperature for 24 h. Constant sheer strain (γ = 1%) and angular frequency (ο = 6 rad/s) were applied in time sweep experiments. Both gels exhibited properties typical of peptide hydrogels, where elasticities (storage modulus G’) are higher compared to the corresponding viscosities (loss modulus G”) (Fig. 1a,b)^23^. In the case of the 1% gel, G’ was more than 6 times stronger than G”, which is a characteristic feature of peptide hydrogels^6^. The value of G’ also indicates moderate mechanical stability.

**Figure 2:**
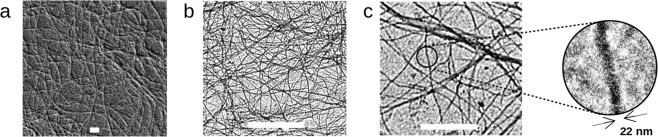
SEM (**a**) and TEM (**b**,**c**) of the 1% SIINFEKL hydrogel, where the sample was imaged without staining on the copper grids. Scale bars: 500 nm (**a**,**c**) and 1 μm (**b**).

The micro- and nanoscale properties of the hydrogel material were studied by electron microscopy and small angle X-ray scattering (SAXS). The presence of fibrillar networks with fibrils on the micrometer range and widths of less than 25 nm is apparent in micrographs of the 1% hydrogel (Fig. 2). SAXS allows for measurement of nanoscale changes in density of a given sample and it is therefore a common technique in evaluation of nanoscale properties of different materials^24^. SAXS measurements of the SIINFEKL hydrogel showed an intensity increase towards small q-values (scattering vector) and a constant intensity towards higher q-values (Fig. SI-2). The data was normalised to time and beam intensity. A decrease of the total X-ray intensity was visible for each subsequent measurement of the sample. This could be caused by increased fibrillisation of the sample, which would decrease the number of scattering bodies. The radius of the fibrils was evaluated from Guinier theory, modified for long rod-like objects with circular cross-section^24,25^. Calculating a weighted mean of the three measurements resulted in a mean radius of the cylindrical objects of 12.3 ± 1.2 nm. The calculated radius correlates well with the diameter of the narrowest long peptide fibrils observed by TEM (22 nm, Fig. 2).

**Figure 3:**
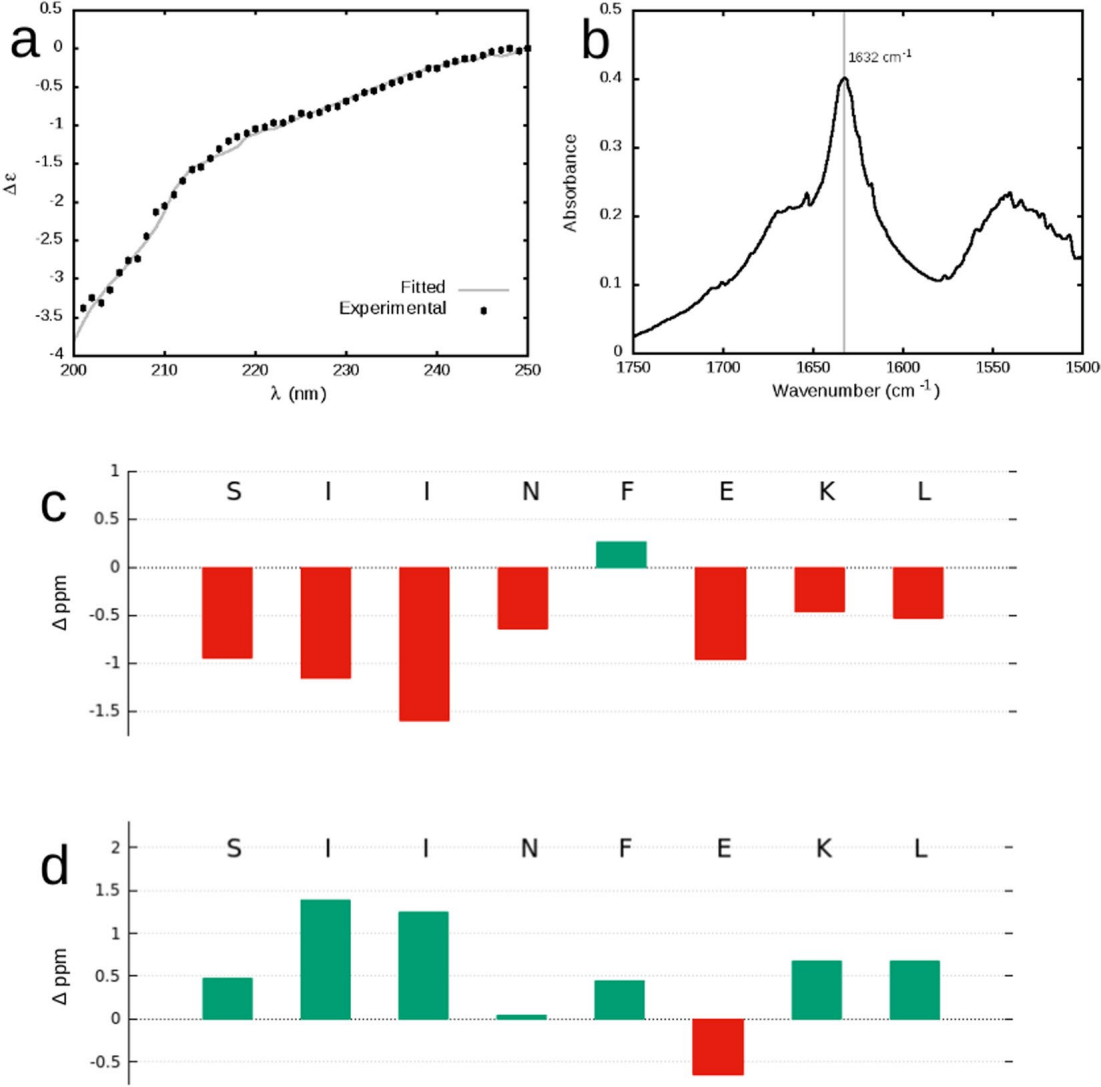
Secondary structure analysis of the peptide hydrogel: CD (**a**) and IR (**b**) spectra of the samples resulting from 1% SIINFEKL hydrogel; differences in C13 C-α (**c**) and C-β (**d**) shifts as compared with random coil shifts, where significant deviations can be seen for the two isoleucine residues.

The peptide conformation within the hydrogel was evaluated by CD and IR spectroscopies. Due to their specific optical activity, secondary structure of different biomolecules can be analysed by use of CD^26^. The corresponding spectra of the peptide hydrogel diluted in water show a combined profile with properties of both a random coil (slope at 200–210 nm, Fig. 3a) and a β-sheet (slope at 210–230 nm). The CD curve was fitted using a β-structure selection algorithm, which predicted 55% of the sample to be in a random coil conformation, 20% anti-parallel β-sheets, and 19% β-turns (fitted curve, Fig. 3a, RMSD = 0.0738)^26^. In conjunction with CD, FTIR is a technique that is often used for evaluation of biomolecular secondary structure. The FTIR spectrum of the SIINFEKL hydrogel was measured following chloride ion exchange in D_2_O. Although broad, a signal spectrum at 1632 cm-1, can be observed (Fig. 3b), which can also be attributed to the amide I band of anti-parallel β-sheets^27^.

The precise secondary arrangement of the individual amino acids can be obtained by use of NMR^28^. NMR spectra of the SIINFEKL hydrogel were recorded from the 5% hydrogel in 1:5 D_2_O/H_2_O. Although hydrogel formation was visible, the NMR spectra did not display any line broadening (Fig. SI-6). By use of TOCSY, it was possible to assign the proton shifts corresponding to the amide, α- and most of the β-hydrogens of the individual amino acids as well as the α- and β-carbons. Most of the identified backbone NMR shifts fell within the range of the random coil conformation with the exception of α- and β-carbons (Fig. 3c,d, respectively) of the isoleucine residues at positions 2 and 3. These shift differences from the random coil structure indicate the β-sheet arrangement for the two isoleucing residues^28^.

In order to evaluate the role of the *N*-terminal isoleucine residues on the gelation properties of the peptide, an analogue with alanine substitutions was prepared (peptide sequence: SAANFEKL). When subjected to conditions, at which SIINFEKL peptide undewent gelation, the solution of the new analogue remained liquid even after a 72 hour incubation at room temperature (Fig. SI-3). Another analogue was prepared, this time with replacement of the *C*-terminal hydrophilic residues by alanines (peptide sequence: SIINFAAL). This analogue proved challenging to dissolve in aqueous buffers due to its increased hydrophobicity, which made it impossible to evaluate the hydrogel forming properties of this peptide.

The propensity of SIINFEKL to form hydrogels has been previously briefly described by Lowenheim and co-workers^29^, who showed that the peptide conjugate can serve a scaffold for neurite outgrowth. However, no analysis of the propertied of the SIINFEKL hydrogel beyond scaffolding was carried out. In this work we show that the morphology of SIINFEKL fibrils is similar to that of other peptide hydrogels, as it consists of a dense network of apparently flexible fibrils but with a rather large diameter of 20–25 nm as opposed to 2–5 nm usually observed for peptide fibrils, as for example for the troponin C peptide fragment VEQLTEEQKNEFKAAFDIFVLGA^11^. TEM micrographs show that the individual SIINFEKL nanofibres further assemble into micro-scale fibres with distinct branched structures. The size and shape of the nanofibres were confirmed by SAXS measurements. Assembly into fibrils is often facilitated by a distinct fold adapted by a portion of the monomer peptide, where β-sheets are by far the most common motifs^6^. Under our experimental conditions the SIINFEKL peptide also adopts a partial β-sheet fold, which is in contrast to the native fold of this peptide within the context of crystalline ovalbumin, where the termini of SIINFEKL are involved in two separate α-helices, the center of the peptide is in a random coil, and no portion of the peptide forms a β-sheet^30^. Our NMR data indicates that only the two *N*-terminal isoleucine residues of the peptide are not in a random coil conformation. Due to their side-chain hydrophobicity, isoleucine residues are known to facilitate self-assembly of short peptides, as is the case for the peptide IIIK that forms stable nanofibers in aqueous solution via β-sheet formation combined with molecular amphiphilicity^31^. The continued assembly of IIIK leads to formation of a soft peptide hydrogel. The fibrillar structure of SIINFEKL assemblies is also likely to be facilitated via such amphilicity, as the N-terminal half of the peptide contains two hydrophobic residues (IleIle), whereas the C-terminal half contains two adjacent hydrophilic amino acids (GluLys).

## Conclusion

As the peptide SIINFEKL is a very well-characterised epitope, it is often used in proof-of-principle studies aimed at demonstrating the efficacies of new adjuvant systems. It has been employed in evaluation of, among others, monophosphoryl lipid A^32^, bacterial membrane vesicles^33^ and HMGB1 peptide^34^. SIINFEKL has also been used to study the immune response to the Bacillus Calmette–Guérin vaccine, which is commonly used against tuberculosis^35^. It is now, however, well-known that peptide hydrogels consisting of nanofibres can themselves act as adjuvants^14,36^, and therefore, our results should caution the use of the SIINFEKL peptide during evaluation of adjuvant efficacies because its ability to form fibrils and a hydrogel.

## Methods

### Peptide synthesis and gel formation

Solid phase peptide synthesis (SPPS) was done using fluorenylmethoxycarbonyl (Fmoc) chemistry on 100 µmol scale. Peptides were deprotected and cleaved in a mixture of trifluoroacetic acid, triisopropylsilane and water (38: 1: 1) for 2 hours at room temperature. The peptide was precipitated with diethyl ether and centrifuged. After washing twice with ether, the precipitated peptide was dissolved in water and lyophilised. The peptide was purified by RP-HPLC using a semi-preparative Kromasil C18 column. Analysis of the purified peptide SIINFEKL (38 mg, 39% yield), was carried out using an analytical Kromasil C4 column with a gradient of 5% buffer B (acetonitrile + 0.08% TFA) in buffer A (water + 0.1% TFA) to 65% B in A over 20 min at 1 mL/min and UV measurement at 214 nm (Fig. SI-1). Peptides SAANFEKL (Fig. SI-4) and SIINFAKL (Fig. SI-5) were synthesised and analysed in a similar manner.

Hydrogel formation was monitored visually as a function of pH, peptide and buffer concentration (Table SI-2). In the standard gelation procedure, water was added to the peptide and the resulting mixture mixed until the sample assumed a homogeneous consistency. Gelation was apparent minutes after dissolving, with final pH 4.2. The pH was then adjusted to 7 by adding aliquots of an appropriate NaOH aqueous solution and the volume was adjusted to give the target final concentration. The solutions were allowed to stand at room temperature overnight. Similar experiments were carried out at a fixed peptide concentration of 1 wt% and samples were incubated at different temperatures.

### Rheology

The rheological measurements were performed on a stress-controlled rheometer (TA Instruments HR2) fitted with a 50 mm diameter plate geometry, with a gap of 0.2 mm. The sample was allowed to anneal at 25 °C for 1 hr prior to time-sweep experiments in linear regime for both strain and frequency as discussed in the manuscript.

### Microscopy

For scanning electron microscopy (SEM), peptide hydrogel was applied to a Thermanox™ coverslip and air dried. The coverslips were sputter coated with gold in high vacuum (Bal-Tec SCD 005). SEM images were recorded with Zeiss SEM Supra 55 VP operating at 20 kV.

For transmission electron microscopy (TEM), peptide hydrogel was applied onto carbon coated copper grid and subsequently viewed with Philips CM200 at 200 kV. TEM images were acquired with OriusTM SC600 Gatan CCD camera.

### Circular Dichroism

CD spectra were recorded on peptide solutions at 0.5% by weight in water using a 1 mm quartz cuvette. Solutions had been incubated at room temperature for a minimum of 24 h. Scans were performed in 1 nm increments with 3 s scans at 20 °C and averaged over 5 scans.

### FTIR

FTIR-ATR was recorded on peptide solutions at 1% by weight in D2O using Agilent Cary 630 with a single bounce diamond ATR-cell and potassium-bromide optics. The spectral resolution was set to 2 cm^-1^, with zero filling factor 2, which resulted in a formal resolution of 0.46892 cm^-1^.

### SAXS

SAXS measurements were performed using X-rays from a Nanostar (Bruker AXS) system, operating at λ = 0.1542 nm (CuKα-radiation) and equipped with a two-dimensional detector (Våntec 2000). X-ray patterns were radially integrated to obtain the scattering intensity of the peptide in dependence on the scattering vector q = (4π/λ) sinθ, with 2θ being the scattering vector. The samples were filled into capillaries and the solvent sample was subtracted as background from the peptide sample dissolved in the solvent. Sample measurement time was 3, 6 and 12 hours.

### NMR

NMR spectra were acquired with a 700 MHz Bruker Avance III HD NMR spectrometer on peptide sample dissolved in 20% D_2_O in H_2_O at 5 mg/ml. For H-H Total Correlation Spectroscopy (TOCSY) 16 transients were collected using 16 dummy scans with spectral width of 10 ppm in both dimensions.

## Supplementary information

Supporting information

## Data Availability

Data generated or analysed during this study are included in the main manuscript file and its supporting information. Additional NMR and SAXS data generated during and analysed during this study are available from the corresponding author on reasonable request.
