# Supplementary material for: Ovalbumin Epitope SIINFEKL Self-Assembles into a Supramolecular Hydrogel
Source: Sci Rep. 2019 Feb 25;9:2696. doi: 10.1038/s41598-019-39148-8 (PMC6390181; doi:10.1038/s41598-019-39148-8)
Supplement: Supplementary file 1 — Supporting information [file 41598_2019_39148_MOESM1_ESM.pdf]

Ovalbumin Epitope SIINFEKL Self-Assembles into a Supramolecular Hydrogel

Supporting Information

Meder Kamalov,[a] Hanspeter Kählig,[b] Christian Rentenberger,[c] Alexander Müllner,[d] Herwig Peterlik[d] and Christian F.W. Becker[a]

a. Institute of Biological Chemistry, Faculty of Chemistry, University of Vienna

b. Institute of Organic Chemistry, Faculty of Chemistry, University of Vienna

c. Physics of Nanostructured Materials, Faculty of Physics, University of Vienna

d. Dynamics of Condensed Systems, Faculty of Physics, University of Vienna

Figures

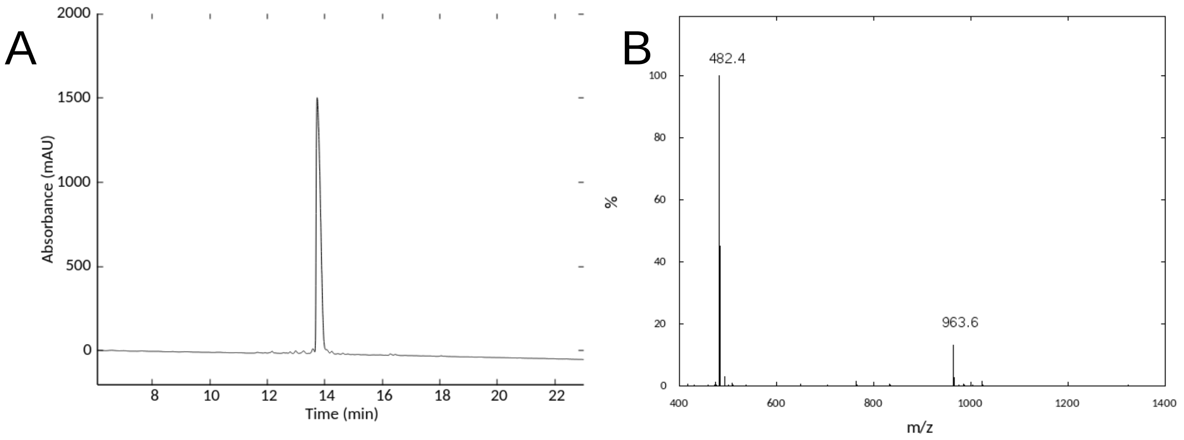

Figure SI-1. LC (A) and MS (B) of the purified peptide SIINFEKL. Calculated $[M+1H]^{1+}$ = 963.5, $[M+2H]^{2+}$= 482.4

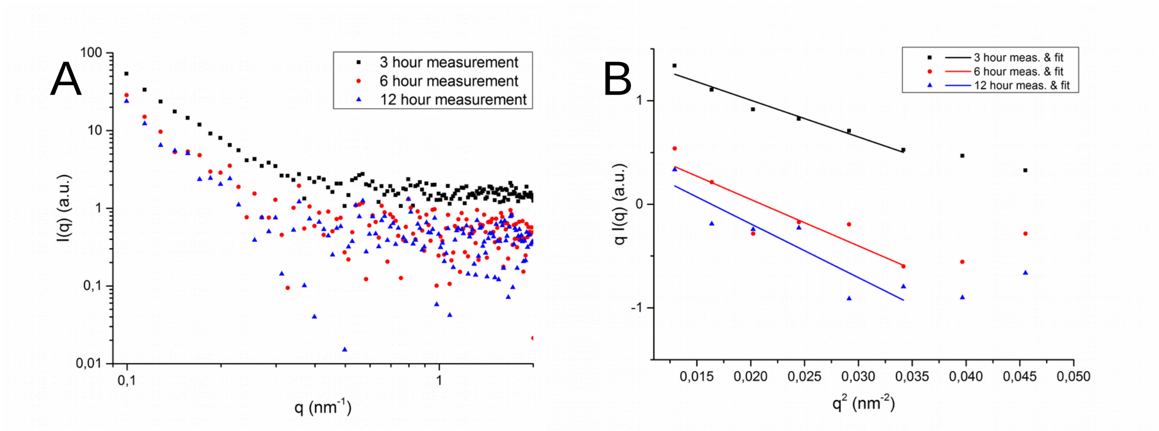

Figure SI-2. SAXS measurements at different time-points (A) and Guinier fit of the respective samples (B).[1]

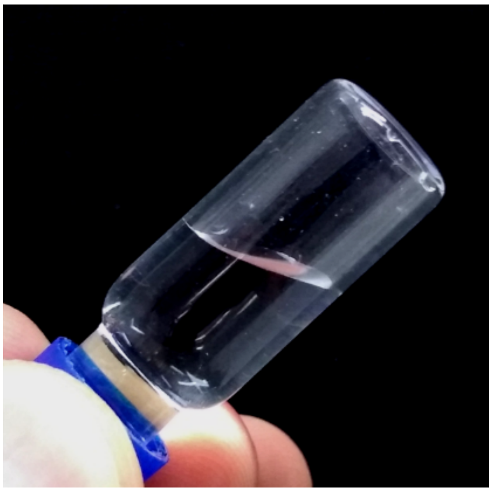

Figure SI-3. 10 mg/ml solution of SAANFEKL in water following 72 h room temperature incubation

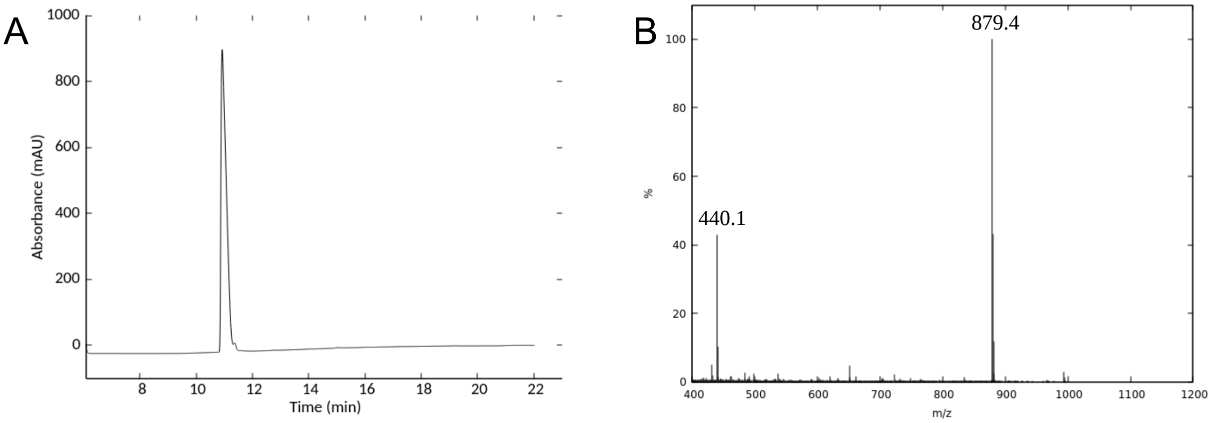

Figure SI-4. LC (A) and MS (B) of the purified peptide SAANFEKL. Calculated $[M+1H]^{1+}$ = 880.0, $[M+2H]^{2+}$= 440.5

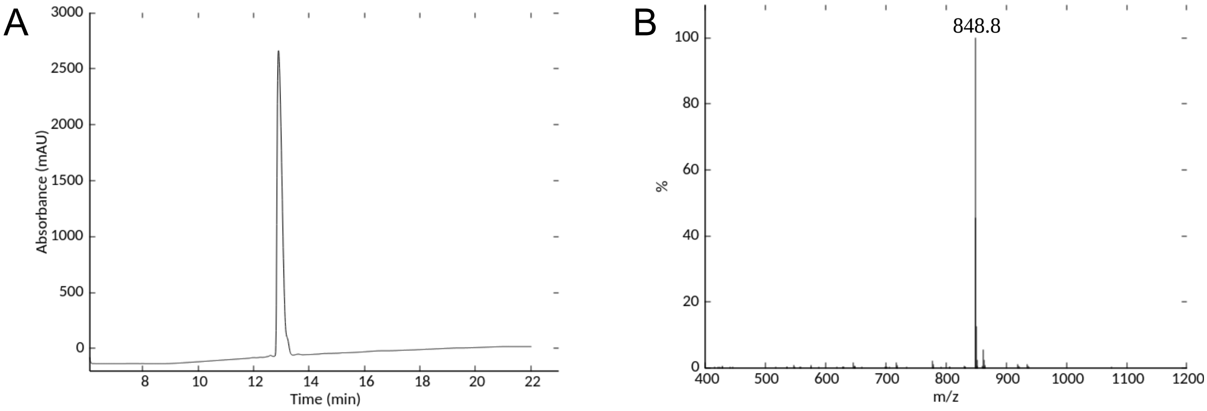

Figure SI-5. LC (A) and MS (B) of the purified peptide SIINFAAL. Calculated $[M+1H]^{1+}$ = 849.0

Table SI-1. NMR Data

Residue	Species	^{1}H (ppm)	$J_{H,H}$ (Hz)	^{13}C (ppm)	^{15}N (ppm)
Ser 1	CO	-	-	170.59	
	NH2				
	CH	4.173	d 4.2 / d 6.1	57.35	
	CH2	3.982	d 12.2 / d 4.2	63.17	
		3.909	d 12.2 / d 6.1		
Ile 2	CO	-	-	175.96	
	NH	8.579	d 7.4		121.57
	CH	4.235	t 7.6	61.44	
	CH	1.832	m	38.89	
	CH3	0.877	d 6.7	17.46	
	CH2	1.458	m	27.32	
		1.177	m		
	CH3	0.862	t 7.5	12.96	
Ile 3	CO	-	-	a 175.50	
	NH	8.243	d 7.9		125.33
	CH	4.097	t 8.1	60.98	
	CH	1.746	m	38.74	
	CH3	0.732	d 6.8	17.38	
	CH2	1.422	m	27.32	
		1.119	m		
	CH3	0.825	t 7.4	12.69	
Asn 4	CO	-	-	174.73	
	NH	8.403	d 7.7		123.31
	CH	4.681	d 6.5 / d 7.6 / d 7.7	52.96	
	CH2	2.757	d 15.6 / d 6.5	39.05	
		2.671	d 15.6 / d 7.6		
	CO	-	-	176.98	
	NH2	7.563, 6.862	s		112.54

Phe 5	CO	-	-	a 175.52	
	NH	8.228	d 7.1		121.45
	CH	4.545	d 6.3 / d 8.4 / d	58.16	
	CH2	3.129	d 14.0 / d 6.3	39.48	
		3.028	d 14.0 / d 8.4		
	C	-	-	138.81	
	CH	7.244	m	131.86	
	CH	7.354	m	131.55	
	CH	7.305	m	130.01	
Glu 6	CO	-	m	175.23	
	NH	8.168	d 7.2		121.86
	CH	4.282	m	55.73	
	CH2	2.03	m	29.05	
		1.916	m		
	CH2	2.358	m	32.92	
	CO	-	-	180.1	
Lys 7	CO	-	m	175.85	
	NH	8.208	d 7.2		123.03
	CH	4.282	m	56.23	
	CH2	1.842	m	32.97	
		1.731			
	CH2	1.433	m	24.66	
	CH2	1.69	m	29.17	
	CH2	3	m	42.22	
	NH2	7.525	br		
Leu 8	CO	-	m	180.42	
	NH	8.189	d 7.8		127.1
	CH	4.318	m	55.17	
	CH2	1.623	m	42.57	
	CH	1.616	m	27.18	
	CH3	0.913	d 6.0	25.08	
	CH3	0.858	d 6.1	23.3	

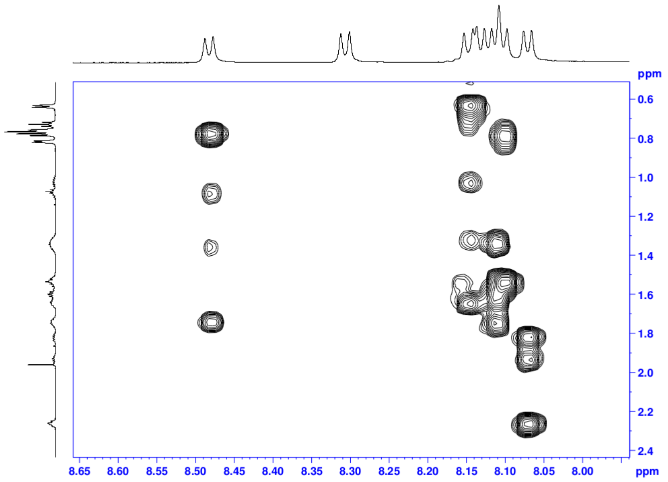

Figure SI-6. Portion of the TOCSY NMR spectrum showing correlation between amide and backbone protons.

Table SI-2: SIINFEKL gel conditions

Buffer	Concentration	pH	Gel
DI Water	2 mg/ml	7	No
DI Water	5 mg/ml	7	Yes (after 2h incubation)
DI Water	10 mg/ml	7	Yes
DI Water	15 mg/ml	7	Precipitate
10 mM potassium phosphate	10 mg/ml	7	Yes
25 mM potassium phosphate	10 mg/ml	7	Yes
25 mM potassium phosphate	10 mg/ml	4	Yes
25 mM potassium phosphate	10 mg/ml	9	Precipitate
50 mM potassium phosphate	10 mg/ml	7	Yes
100 mM potassium phosphate	10 mg/ml	7	Precipitate

References

1. O. Glatter and O. Kratky, *Small angle X-ray scattering*, Academic Press, Cambridge, 1982
